# Molecular markers of cell adhesion in ameloblastomas. An update

**DOI:** 10.4317/medoral.19071

**Published:** 2013-08-29

**Authors:** Rogelio González-González, Nelly Molina-Frechero, Pablo Damian-Matsumura, Ronell Bologna-Molina

**Affiliations:** 1 DDS, MSc. Research Department, School of Dentistry, Universidad Juárez del Estado de Durango (UJED), Durango, Mexico and Doctorado en Ciencias Biológicas y de la Salud, Universidad Autónoma Metropolitana. Mexico City, Mexico; 2 DDS, PhD. Health Care Department, Universidad Autónoma Metropolitana, Xochimilco. Mexico City, Mexico; 3 MD, PhD. Department of Biology of Reproduction, Universidad Autónoma Metropolitana, Iztapalapa. Mexico City, Mexico; 4 DDS, PhD. Research Department, School of Dentistry, Universidad Juárez del Estado de Durango (UJED), Durango, México and School of Dentistry, Universidad de la República (UDELAR), Montevideo, Uruguay

## Abstract

Ameloblastoma is the most common odontogenic tumor of epithelial origin, and though it is of a benign nature, it frequently infiltrates the bone, has a high rate of recurrence and could potentially become malignant. Cellular adhesion potentially plays an important role in the manifestation of these characteristics and in the tumor biology of ameloblastomas. Losses of cell-cell and extracellular matrix adhesion and cohesion are among the first events that occur in the invasion and growth of tumors of epithelial origin. The present review includes a description of the molecules that are involved in cell adhesion as reported for various types of ameloblastomas and discusses the possible roles of these molecules in the biological behaviors of this odontogenic tumor. Knowledge of the complex mechanisms in which these molecules play a role is critical for the research and discovery of future therapeutic targets.

** Key words:**Ameloblastoma, cellular adhesion, molecular markers, cell-cell adhesion, extracellular matrix-cell adhesion.

## Introduction

Cell adhesion molecules (CAMs) are found at the surfaces of all cells, where they allow dynamic processes to take place during tissue morphogenesis and during the development and maintenance of adult tissues. These molecules are essential to the maintenance of stratified epithelial structures because they participate in the processes of cell renewal and mobility and are fundamental to cell junctions and extracellular matrix (ECM) interactions (1). More than 50 CAMs have been identified; and form large superfamilies. Most proteins connect the extracellular and intracellular environments, as they are comprised of structures that bind junctions between the cytoskeleton and the ECM or between cells. Through these structures, the cell executes signaling and signal transduction functions and regulates processes such as cell division, migration and differentiation. These structures also have important functions during the development of dental organs. Although the function of CAMs in dental development is not currently well understood, it is known that CAMs participate in cell-cell and ECM-cell interactions during histomorphogenesis in the different stages of odontogenesis (2,3). Cell adhesion interactions are strictly regulated, which involves numerous cell signaling pathways that are responsible for regulating the expression of cell adhesion molecules and the duration of adhesive contacts. This control ensures the integrity and stability of morphogenesis and tissue maintenance. However, many of these processes are dysregulated in various tumors, which permits tumor progression, recurrence, invasion and metastasis. The loss of cell adhesion allows neoplastic cells to escape their places of origin, degrade the ECM, acquire motility, invade tissues and possibly cause metastasis (2,4). Ameloblastomas (AMs) are locally infiltrative benign tumors that rarely convert to malignancy, though they are locally invasive and carry a high risk of recurrence (3,4).

The World Health Organization (WHO) has classified this neoplasia into the following variants: solid/multicystic ameloblastoma (SMA), unicystic ameloblastoma (UA), desmoplastic ameloblastoma (DA) and peripheral ameloblastoma (PA), as well as malignant counterparts such as malignant ameloblastoma (MA) and ameloblastic carcinoma (AC) (5). A tumoral invasion of neoplastic cells into surrounding healthy tissues is a characteristic that promotes progression and recurrence. Therefore, the infiltrative property of AMs is a characteristic that is potentially associated with alterations in tumor cell CAMs (6). The molecular mechanisms of tumor invasion have not been clearly defined; consequently, our study focuses on a review of CAMs to better understand the relationships that exist between these molecules and AM tumor cell invasion ( Table 1).

**Table 1:**
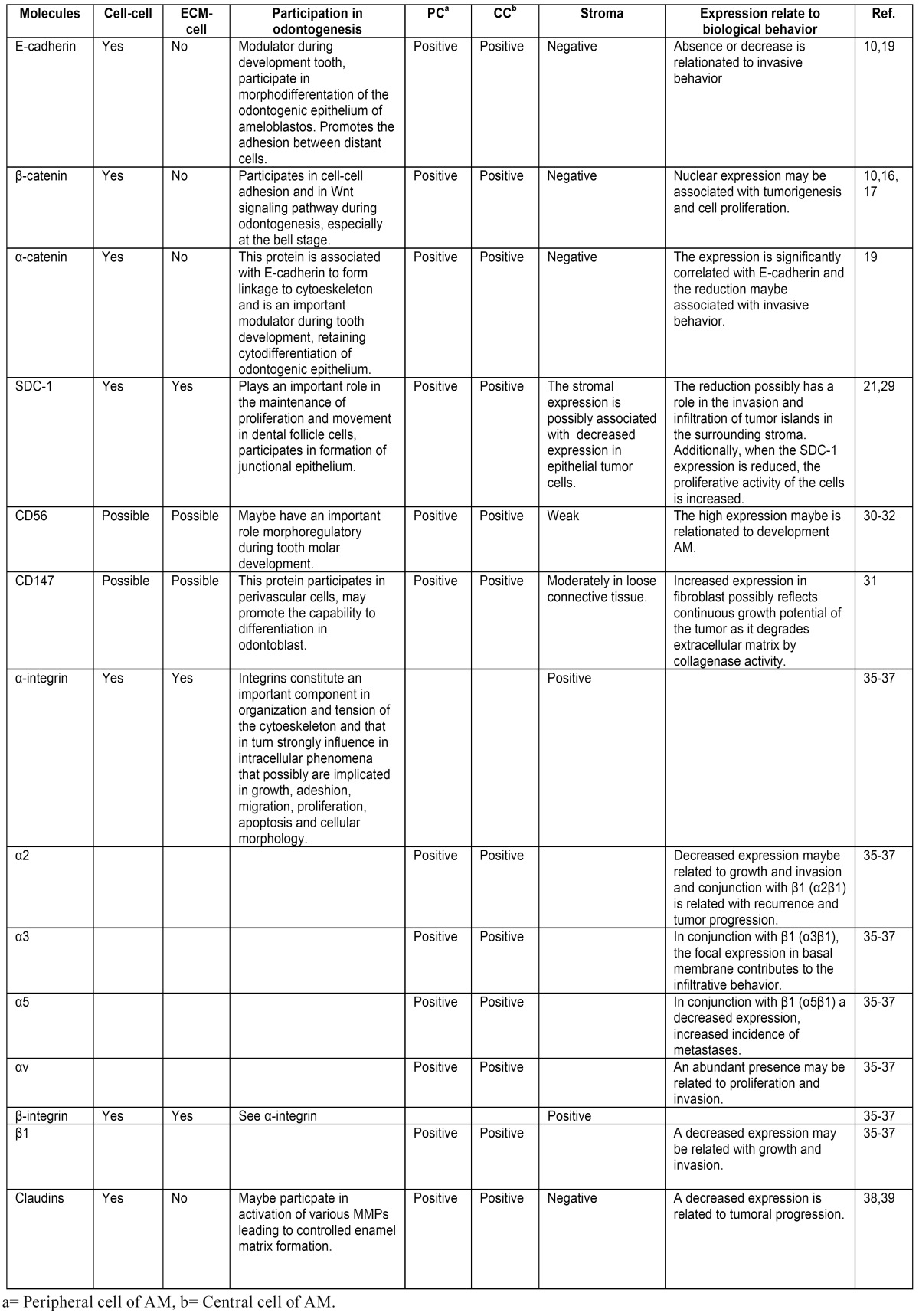
Expression of different cell adhesion molecules and their participation in: odontogenesis, tumorigenesis and biological behavior of AM.

## Cell Adhesion Molecules

-Cadherins

**Figure 1:**
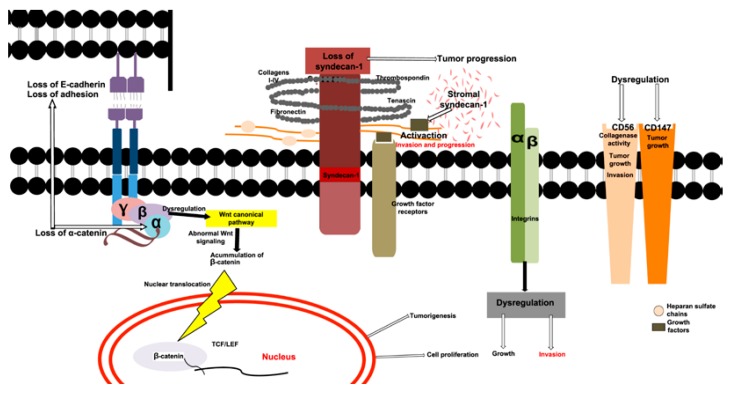
Schematic representation of the roles of CAMs in cell-cell and cell-ECM junctions in an AM neoplastic cell. Loss of E-cadherin and α-catenin are indicative of invasion and possibly metastasis. A dysregulation of β-catenin affects the Wnt canonical pathway, this alteration maybe is associated with accumulation of β-catenin in cytoplasm, this accumulation causes translocation of β-catenin to the nucleus, activates the transcription factors (TCF/LEF) that promote cell proliferation and tumorigenesis. The loss of SDC-1 in epithelial cells is associated with tumor progression and accumulation of SDC-1 in the stroma, this accumulation possibly is associated to activation of growth factors heparin binding, these factors possibly are involved in the invasion and tumoral progression. A dysregulation of integrins are relationated to growth and tumor invasion. Possibly a dysregulation of CD56 and CD147 are related to growth and tumor invasion.

Cadherins are CAMs that are expressed on cell membranes in the adherens junction and that have the ability to communicate with different intracellular controls (7). Cadherins are classified as follows: E (epithelial), P (placenta), M (muscle), N (nerve), B (brain) and R (retina) cadherins, and these molecules can combine with α-, β- and γ-catenin (7). Cytoplasmic controls connect actin filaments to the cell cytoskeleton and, in this way, can regulate the adhesive capacities of cadherins (8). Studies of the functions of cadherins in AMs have been motivated by the presence of E, P, and N cadherins in the enamel and because of the roles that cadherins play in odontogenesis (9,10). One such study was conducted by Alvez-Pereira et al. (10), in which immunohistochemistry was used to discover the strong expression of E-cadherin on cells that were similar to the stellate reticulum of AMs. This result suggested that high concentrations of this molecule exist in specific sites of the stellate reticulum, which promote adhesion in distant cells. Furthermore, various authors have studied the E-cadherin promoter hypermethylation as an important factor in the malignant transformations of various carcinomas (11). From these references, it is apparent that previous studies have attempted to associate E-cadherin promoter hypermethylation with malignant transformations in AMs. However, such studies have concluded that E-cadherin promoter hypermethylation in AMs might not be associated with tumor progression (12,13). As mentioned previously, E-cadherin is an important regulator of cell adhesion; therefore, the loss of E-cadherin could be associated with tumor advancement in AMs. The maintenance of E-cadherin expression in well-differentiated tumors can be interpreted as the conservation of adhesion between tumor cells and the tissue architecture, which is associated with a better patient prognosis. However, in poorly differentiated tumors, E-cadherin expression was diminished, which suggests a loss of adhesions between the tumor-forming cells (Fig. 1). This result could indicate that the malignant neoplasms are capable of spreading by invasion and metastasis, which correlates with a poor patient prognosis (14).

-β-Catenin

β-Catenin is a protein that participates in cell-cell adhesions and is important for the regulation of adhesion complexes. Along with cadherin subunits, this 92-kDa protein forms a protein complex that participates in the adherent junctions that are necessary for cell adhesion and growth regulation. β-catenin plays an important role in the Wnt canonical pathway, and after its interaction with Wnt, acquires the ability to control fundamental mechanisms of cell proliferation, cell polarity and cell fate determination during embryonic development and adult tissue homeostasis (15). The dysregulation of β-catenin is related to the genesis of numerous malignant neoplasias, indicating an important role for this molecule in tumor progression (16). Various immunohistochemistry analyses of β-catenin in AMs have indicated that β-catenin is expressed in the cytoplasm and cell membrane, as well as in columnar epithelial cells that resemble the stellate reticulum. However, nuclear expression of β-catenin was observed mainly in the SMA variants and in odontogenic carcinomas (10,16,17). This finding is important because the nuclear expression or nuclear accumulation of β-catenin might indicate abnormal Wnt signaling, which could be related to tumorigenesis and cell proliferation in AMs (16-18) (Fig. 1).

Therefore, the nuclear accumulation of β-catenin might be related to AM variants that exhibit aggressive, invasive and recurrent behaviors and might also be associated with increased cell proliferation (13,16,17).

-α-Catenin

α-Catenin is a protein that associates with E-cadherin; together, these proteins form links with the cytoskeleton proteins and allow for the maintenance of epithelial tissues, thus playing an important role in organogenesis and morphogenesis. As such, the loss of α-catenin expression might be associated with dedifferentiation, invasion and metastasis (17). The expression patterns of E-cadherin and α-catenin in AMs are similar to those in the enamel organ that maintains odontogenic epithelium cytodifferentiation (19). Kumamoto and Ooya (19) have conducted a study of E-cadherin and α-catenin, concluding that the expression of these two molecules could be indicative of conserved cell-cell adhesion. Additionally, E-cadherin and α-catenin expression are uniformly distributed within the cell-cell boundary, which indicates conservation of cell adhesion functions. Studies have associated the expression of these two proteins with dedifferentiation, invasion and metastatic potential (18,19). In malignant neoplasias, an important reduction in the expression of both proteins was observed, which agreed with studies that demonstrated evidence of MA intraosseous invasion and lymphatic metastasis and reported a prominent reduction in the expression of E-cadherin and α-catenin (19) (Fig. 1).

-Syndecans

**Figure 2:**
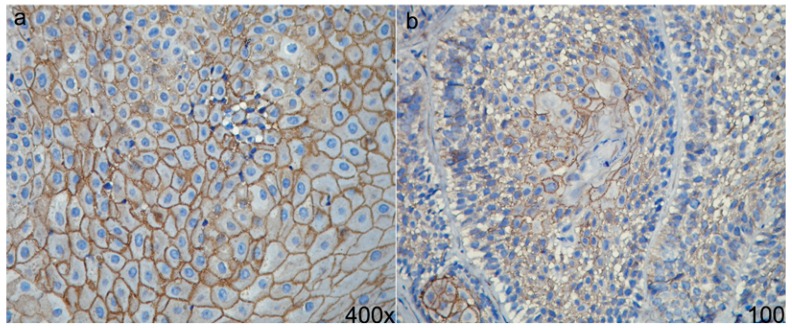
a) Epithelial expression of SDC-1 in normal oral mucosa, this expression is observed in surrounding of epithelial cells, and is indicative that cells have an adequate cell-cell adhesion. b) Epithelial expression of SDC-1 in acanthomatous variant of SMA, this expression is associated with the maintenance of epithelial morphology, anchorage-dependent growth and inhibition of invasiveness.

Syndecans form the largest family of heparin sulfate proteoglycans, which are expressed on the surfaces of all adherent cells, as well as on many non-adherent cells. In mammals, syndecans consist of a family of four members, and each is coded from a different gene (20). The most important and most well studied of these members is Syndecan-1 (SDC-1), which is mainly ex-pressed on epithelial and plasma cells (20). SDC-1 participates in cell-cell and ECM-cell adhesions and is considered an im-portant structural maintenance protein, along with other molecules such as collagens I, II, III, IV; fibronectin; thrombospondin; and tenascin. SDC-1 co-participates with growth factors such as basic fibroblast growth factor (bFGF), vascular endothelial growth factor VEGF and epidermal growth factor (EGF), among others (21-23). SDC-1 is mainly located on the basolateral surfaces of simple epithelial and surrounding stratified epithelial cells. Although SDC-1 is not present on the majority of mesenchymal cells in mature tissues, its expression is observed in small quantities within mesenchymatous cells in culture (23,24). SDC-1 gene expression, ranging from overexpression to complete absence, has been studied in various types of carcinomas (25-29). In squamous cell carcinomas, the loss of this protein is commonly associated with greater levels of invasion and metastasis. In the particular case of AMs, differential expression of SDC-1 has been observed in diverse histological types, which suggests that this protein participates in the biological behavior of these tumors, (Fig. 2). Various studies have suggested that SDC-1 gene expression in AMs is related to the AM variant or histological type (28,29). Immunohistochemistry techniques have revealed increased SDC-1 gene expression in the UA, DA and PA variants and reduced expression in SMA, recurrent SMA and AC. Therefore, it has been suggested that the loss of SDC-1 gene expression is related to tumor progression and that stromal gene expression of SDC-1 might be associated with the activation of various growth factors that are related to invasion, progression and metastasis. In this way, SDC-1 plays an important role in carcinogenesis and the interosseous invasion of AMs. Studies conducted by Otaibi et al. (21) demonstrated increased stromal SDC-1 gene expression in the SMA and AM variants, with body extensions to the mandibular nerve branches, and in variants that crossed the cell midline. Therefore, it is possible that the stromal expression of SDC-1 is indicative of more aggressive and recurrent AM variants (Fig. 1).

-N-CAM (CD56) and neurothelin (CD147)

Various surface antigens are involved in the aggregation, organization and metastatic nature of tumor cells. CD56 is a transmembrane protein that belongs to the immunoglobulin superfamily and is a specific marker for natural killer (NK) cells. CD56 plays an important morphoregulatory role through an interaction between the epithelium and mesenchymal cells that are derived from neural crest cells. It is possible that this molecule is essential to the formation of the basic structures of teeth and periodontal tissue (30,31). CD147, a transmembrane protein that belongs to the immunoglobulin superfamily, has been described as a soluble factor that acts in cell adhesion and collagenase activity stimulation in fibroblasts (31,32). CD56 and CD147 are similar molecules that might contribute to cell-cell and ECM-cell adhesions and can participate in the aggregation and migration of tumor cells (31). In AMs, CD56 expression is observed in the peripheral cells at tumor sites and is absent in the stellate reticulum, whereas CD147 is expressed with greater intensity in the peripheral cells of tumor sites. Cairns et al. (32) observed that the expression of CD147 was limited to the cell membranes of columnar ameloblast-like cells and to the periphery of epithelial cell nests and SMA epithelial chords. From these molecular characteristics, it can be hypothesized that CD147 and CD56 expression in AM epithelial cells might indicate an ability to degrade the ECM and to promote continuous tumor growth and invasion (31) (Fig. 1).

-Integrins

Various types of cells cannot proliferate without being anchored to ECM substrates. Therefore, ECM proteins play an important role in the interactions between the epithelium and mesenchyme. Such proteins are important components of the cytoskeletal organization and tension and thus can strongly influence intracellular phenomena, such as proliferation and cell differentiation (33). Integrins constitute an important family of transmembrane receptor proteins that bind to cell surfaces and to ECM ligands, where they participate in anchoring to the ECM proteins and in the modulation of multiple molecules that are involved in growth, adhesion, migration, proliferation, apoptosis and cell morphology (34,35). The above data indicate that integrins can activate multiple molecules necessary for cell survival. Therefore, the dysregulation of these molecules might be related to tumor invasion (34). Modolo et al. (35) observed strong expression of the α1, α2, α3, α5, αv, β1, β3 and β4 integrins in follicular, acanthomatous and plexiform SMAs, as well as in luminal UAs. The authors also found that the α3, α5, αv, β1 and β4 integrins were expressed in the acanthomatous areas of acanthomatous SMAs, whereas in luminal UAs, the integrins were predominantly expressed in the basal strata. Moreover, in follicular SMAs, the integrin expression was observed at the peripheral layers of neoplastic follicle basal cells, which suggests that the integrins interacted strongly in the basal membranes. These data suggest that decreases in integrin expression are related to tumor growth and the invasion of neighboring structures (35), which is supported by the lack of integrin expression in metastatic tumors. Nevertheless, α2 integrin expression levels are low in AMs in comparison to those in the dental laminates and dental germ, which might be related to the characteristics of growth and invasion in AMs. These data suggest that in AMs, such effects are caused by the modified repertoire of integrins of neoplastic cells, which regulate anti-adhesive and adhesive events in tumor development and thereby explain the observed differences in expression levels (35-37) (Fig. 1).

-Claudins

Claudins are a family of proteins that participate in tight junctions, they are present in epithelial and endothelial cells and are important to functions of barrier, electric resistance and paracellular ionic selectivity, also participates significantly in embryogenesis and organogenesis, mainly in the epithelial-mesenchymal transition (38). Due to their characteristics, claudins have been studied in various tumors and their absence or overexpression are related to behavior. Bello et al. (38) found in AM an intense immunoreactivity of claudins 1, 4 y 7, principally in stellate reticulum like cells, this increased expression can indicate the effort of these proteins to maintain cell-cell adhesion in where commonly exist formation of microcysts and is considerate that expression of claudins 1, 4 y 7 maybe are related to cystic degeneration. Claudin 1 intense expression is seen in regions of squamous differentiation in AM, this finding is important because claudin 1 is distributed in all squamous epithelia cells and has an important participation in cell junctions, whereby this expression maybe associated with squamous diferentiation of AM (39). In the AC, the expression of claudins 1, 4 y 7 is generally weak or moderate and it can be correlated to aggressive behavior of these carcinomas, remembering the absence or decrease of expression of claudin 7 is related with invasion or bad prognosis of carcinomas (38,39).

Consequently changes in the expression of these proteins maybe related to events of tumoral development of AM.

## Conclusions

Diverse types of molecules and gene alterations affect the development and progression of odontogenic tumors, and these characteristics appear to depend on diverse molecular mechanisms. In AMs, epithelial odontogenic neoplastic cells are influenced by a series of molecular alterations that promote tumor growth and tissue invasion. Of these alterations, the loss or dysregulation of various CAMs is critical to the process, as cell adhesion is fundamental to the biological behaviors of odontogenic tumors. The mechanism that could explain the influence of such molecular alterations in tumor advancement and progression is not yet understood. Although various studies have attempted to explain the biological role of CAMs in AMs, it remains necessary to understand the interactions of these molecules with each other and with the various proteins involved in the proliferative, apoptotic and invasive pathways that are characteristic of AM tumorigenesis. Such an understanding might allow for the development of therapeutic applications that block the cell signaling pathways that are involved in the progression of AMs.
